# Prevalence and predictors of exclusive breastfeeding for the first six months of life among women in Halaba special woreda, Southern Nations, Nationalities and Peoples’ Region/SNNPR/, Ethiopia: a community based cross-sectional study

**DOI:** 10.1186/s13690-015-0098-4

**Published:** 2015-12-01

**Authors:** Asrat Sonko, Amare Worku

**Affiliations:** Public health Integrated Family Health Program/IFHP, P.O. Box 90, Hawassa, Ethiopia; Addis Continental Institute of public health, Addis Abeba, Ethiopia

**Keywords:** Exclusive breastfeeding, Prevalence, Predictor

## Abstract

**Background:**

Exclusive breastfeeding is defined as feeding infants only breast milk, be it directly from breast or expressed, with no addition of any liquid or solids apart from drops or syrups consisting of vitamins, mineral supplements or medicine, and nothing else. Several studies have shown that exclusive breastfeeding for the first six months plays a great role in preventing morbidity and mortality. In Ethiopia, for example, mortality rates of infant and children younger than five years are high. Understanding the prevalence and predictors that influence exclusive breastfeeding in this is crucial to promoting the practice.

**Objectives:**

To assess the prevalence and predictors of exclusive breastfeeding with in the first six months of life among women in Halaby special woreda, SNNPR (Southern Nations, Nationalities and Peoples’ Region) Ethiopia.

**Methods:**

A community-based cross sectional study was conducted between November 2013 and January 2014 among women with infants aged less than 6 months and the sample size was 422 in Halaba special woreda, SNNPR (Southern Nations, Nationalities and Peoples’ Region) Ethiopia. A random sampling technique was used in sampling the study subjects. Structured questionnaire was developed and adopted from EDHS 2011 and WHO standard and that all the variables to be assessed were incorporated. Data was entered and analyzed through using Epi Info 7 for Dos version 3.5.1 and Statistical Package for the Social Sciences (SPSS) software for windows version 20 respectively.

**Results:**

The prevalence of exclusive breastfeeding was (70.5 %) and awareness of exclusive breastfeeding was (92.4 %). Antenatal follow up (AOR/Adjusted odds ratio = 1.9; 95 % CI, 1.05–3.6), postnatal follow up (AOR/Adjusted odds ratio = 2.2; 95 % CI, 1.25–3.87), initiation of breast feeding immediately within one hour (AOR/Adjusted odds ratio =3.8; 95 % CI, 2.4–6.2), attended formal school (AOR Adjusted odds ratio/=1.9;95 % CI,1.1–3.4), advice about exclusive breastfeeding (AOR Adjusted odds ratio/=6.3;95 % CI,1.3–30.9); and awareness about exclusive breastfeeding (AOR Adjusted odds ratio/= 8.2; 95 % CI 3.34–20), have significant association with exclusive breastfeeding.

**Conclusions:**

Strategies that target improving awareness among women at health facility and community level help to improve exclusive breastfeeding. In addition, promotion of EBF for working mothers through working environment recommended.

## Background

Exclusive breastfeeding (EBF) for the first six months of an infant’s life is a cost effective intervention in saving children’s lives and it is recommended by the World Health Organization (WHO) [1, 2]. Exclusive breastfeeding (EBF) means giving only breast milk to the infant, without mixing it with water, other liquids, tea, herbal preparations or food in the first six months of life, with the exception of vitamins, mineral supplements or medicines. It is estimated that, with exclusive breastfeeding (EBF) coverage of 90 %, 13 to 15 % of deaths of children fewer than 5 years could be averted in low and middle income countries [1–5]. Exclusively breastfed infants have been shown to have lower rates of acute respiratory infections and diarrhea, to have better neurodevelopment outcomes and have better physical growth compared to mix-fed or non-breastfed infants. In areas where HIV prevalence is high, especially in sub-Saharan Africa, exclusive breastfeeding (EBF) has been shown to have an added advantage of reducing the rates of mother-to child transmission of HIV (MTCT) [5, 6].

Despite its demonstrated benefits, exclusive breastfeeding (EBF) prevalence and duration in many countries including Ethiopia are lower than the international recommendation of exclusive breastfeeding for the first six months of life which is 90 % [1]. Based on several studies done in Ethiopia, breastfeeding is nearly universal but the proportion of exclusively breastfed children up to 6 months is less than the optimal recommendations. Breastfeeding is very common in Ethiopia, with 98 % of children ever breastfed [7]. WHO recommended that children receive nothing, but exclusive breastfeeding for the first six months of life [1]. Over half (52 %) of children under six months in Ethiopia are being exclusively breastfed [7].

In Ethiopia, for example, mortality rates of infant and children younger than five years are high. The main causes of mortality for infants under the age of 6 months are Pneumonia, neonatal conditions, diarrhea, malaria and measles. However, also a large portion of infants are not exclusively breastfed according to the infant feeding recommendations. Understanding the prevalence and predictors that influence exclusive breastfeeding is crucial to promoting the practice [8]. This study was implemented to identify prevalence and predictors of exclusive breastfeeding among women in Halaba special woreda, SNNPR (Southern Nations, Nationalities and Peoples’ Region) Ethiopia.

### Objectives

#### General objective

- ✓ To assess the prevalence and its predictors of exclusive breastfeeding practice with in the first six months of life among women in Halaba special woreda SNNPR (Southern Nations, Nationalities and Peoples’ Region) Ethiopia.

#### Specific objectives

- ✓ To assess the prevalence of exclusive breastfeeding practice with in the first six months of life among women in Halaba special woreda, SNNPR (Southern Nations, Nationalities and Peoples’ Region) Ethiopia
- ✓ To determine the predictors of exclusive breastfeeding practice with in the first six months of life among women in Halaba special woreda, SNNPR (Southern Nations, Nationalities and Peoples’ Region) Ethiopia

## Methods

### Study design

A community based cross-sectional study among mothers with children less than six months of age was held from November, 2013 to January, 2014 in Halaba special woreda, SNNPR (Southern Nations, Nationalities and Peoples’ Region) Ethiopia.

### Study setting and participants

The woreda is one of the 4 special woreda which is found in SNNPR (Southern Nations, Nationalities and Peoples’ Region). The woreda has 79 rural kebeles and 2 urban kebeles. According to the woreda annual report, the total population of the special woreda is estimated about 296,540 of which female 151,235 and male is 145,305. Number of house hold, urban population and rural population is 60,518, 35,595 and 260,945respectively. The number of under one children is 9667, under one surviving infant is 9104, under five children is 46,260, women with child bearing age 15–49 is 69,094 and the number of pregnant mother is about 10,675 [29].

The source of populations for the study was all mothers of children aged less than six months in Halaba special woreda. Therefore, the required sample sizes from each kebeles was calculated from the sampling frame produced for each kebele according to the size of the population (was proportionate sample size allocation).

### Flow diagram (sampling procedure)

A random sampling technique was used in sampling the study subjects. All the 81 kebeles were included in the sampling frame and out of which randomly selected six kebeles using the random number table proportional to the size of kebeles.

- Inclusion Criteria

The study subject was mother to child paired groups, children aged less than six months of age who have mothers were included in the study.

- Exclusion Criteria

Who are not willing mothers, mothers too sick or mentally not stable to respond to questions were excluded from the study.

### Measurements/data sources

The source of populations for the study was all mothers of children aged less than six months in Halaba special woreda. The proportion of children less than six months of age constitutes 4448 of the total population. Therefore, the required sample sizes from each kebeles was calculated from the sampling frame produced for each kebele according to the size of the population (was proportionate sample size allocation).

The sample size required for the study was calculated using the formula to estimate a single and double population proportions.

For first objective:

$$ \mathrm{n}=\frac{\left[{\left(\mathrm{Z}\upalpha /2\right)}^2\mathrm{p}\left(1-\mathrm{p}\right)\right]}{{\mathrm{d}}^2}=\frac{(1.96)^2\times 0.52\left(1-0.52\right)}{(0.05)^2}=384+\left(384\times 10\%\right)=422 $$

Assumption: Based on the previous EDHS 2011, the prevalence of EBF is 52 % [7].

Where: *n* = required sample sizes

10 % = non -respondent rate

Zα/2 = critical value for normal distribution at 95 % confidence interval which equals to 1.96 (Z-Value at alpha =0.05)

*P* = established prevalence from EDHS 2011 (prevalence of exclusive breast feeding = 52 %) [7].

d = an absolute precision (margin of error) =5 %

For the second objective: Used the following formula or Epi info 7 version 3.5.1

Let

$$ \mathrm{P}=\frac{\mathrm{P}1+rP}{1+r} $$

$$ \mathrm{n}=\frac{{\left(Z,\alpha, /, 2,\sqrt{\left(1+1/r\right)},, \mathrm{P},\left(1-\mathrm{P}\right),+,Z,\beta, \sqrt{\mathrm{P}1\left(1-\mathrm{P}1\right)+\frac{\mathrm{P}2\left(1-\mathrm{P}2\right)}{r}}\right)}^2}{{\left(\mathrm{P}1-\mathrm{P}2\right)}^2} $$

Assumptions: 1. Based on the previous study Pre-lacteal feeding considered as important predictor of exclusive breast feeding [8].

41.9 % of mothers with pre-lacteal feeding and NOT practicing EBF and

A. Mothers with pre-lacteal feeding and practicing EBF/Odds of EBF (OR = 2).
  80 % power
  95 % CI
  10 % non-response rate

Assumptions: 2. Based on the previous study place of delivery considered as important predictors of exclusive breastfeeding [8].

B. 32.1 % of mothers delivering at health facility and NOT practicing EBF and
C. Mothers delivering at health facilities and practicing EBF/Odds of EBF (OR = 4.5)
  80 % power
  95 % CI
  10 % non-response rate

Assumptions: 3. Based on the previous study type of delivery/caesarean section considered as important predictors of exclusive breast feeding [8].

D. 40 % of mothers type of delivery caesarean section and NOT practicing EBF and
E. Mothers type of delivery caesarean section and practicing EBF/Odds of EBF (OR = 2.25)
  80 % power
  95 % CI
  10 % non-response rate

Therefore for adequacy, representativeness and to meet the objective the first sample size which was 422 implemented for mother to child paired subjects.

Structured questionnaire was developed and adopted from EDHS 2011 like educational status of the mothers, economical status, religion, mother occupation, health care seeking behavior, exclusive breast feeding practice, and WHO standard included recall methods with in the last24 h and that all the variables to be assessed were incorporated [2]. Questionnaires were developed first in English then were translated to Amharic version. The data collection was conducted by the health professional from the existing health system that to easily familiarized with the tool while conducting the training and actual data collection. Women were first informed about the study and its aim, and those agreeing to participate were given a written consent. Face-to-face interviews were then conducted at participant’s home. Besides this, the investigator carefully entered data and thoroughly cleaned it before starting the analysis.

### Operational definitions

**Early initiation of breastfeeding**: Proportion of children born in the last 24 months who were put to the breast within one hour of birth.

**Exclusive breast-feeding**: Proportion of infants 0–6 months of age who are fed exclusively with breast milk.

Applies to the infants, have received only breast-milk from his/her mother, and no other liquids or solids with the exception of drops or syrup consisting of vitamins, mineral supplements or medicines.

**Predominant breast-feeding**: in addition to breast milk, the infant may be receiving water-based drinks including plain water, tea, and soft drink; no food based fluid or milk will be allowed within the preceding 24 h.

**Full breast-feeding**: breast-feeding exclusively or predominantly.

**Any breast-feeding**: breast-feeding exclusively, predominantly or with any supplements, including milk and solids.

**Early cessation of breast-feeding**: Complete cessation of breast-feeding before the child’s first birthday.

**Pre-lacteal feeding**: Giving anything else for the infant within 6 months age.

**Still breastfeeding**: continuous exclusive breastfeeding is based on a cross section of children in a given age range, in this case children from birth to just 6. Indicators for assessing infant and young child feeding practices under 6 months of age.

### Statistical methods

Data was entered through Epi Info 7 for Dos version 3.5.1 then exported to Statistical Package for the Social Sciences (SPSS) software windows version 20 for analysis. Descriptive statistics was to determine the prevalence of exclusive breast feeding. Accordingly to calculate the prevalence of exclusive breast feeding, the respondents for exclusive breast feeding practice considered as numerators (n) and the partner or the eligible to be participated as denumerators (N). Proportion was compared by exclusive breast feeding using correlation of independent variables. To identify associated factors, first a bivariate logistic regression was performed for each independent variable with the outcome of exclusive breast feeding. Finally, multivariable logistic regression was implemented to determine independent predictors of exclusive breast feeding. All tests were *P* < 0.05 considered as statistically significant.

### Variables

The framework can be used to generate hypotheses about factors affecting exclusive breastfeeding and the types of interventions that might be used to address them.

**Individual level factors**: relate directly to the mother, infant, and the ‘mother-infant dyad’. They include the mother’s intention to breastfeed, her knowledge, skills and parenting experience, the birth experience, health and risk status of mothers and infants, and the nature of early interaction between mother and infant.

**Group level factors**: are the attributes of the environments in which mothers and infants find themselves, the attributes that enable mothers to breastfeed. Environments with a direct influence on mothers and infants include: the hospital and health facilities environment, in which practices and procedures such as infants routinely rooming-in with mothers to allow demand feeding, postpartum skin-to-skin contact and providing professional support with breastfeeding technique difficulties influence the early feeding experience and the follow-up care and support, the home and peer environment, where physical and social factors such as size of household, parity, family circumstances, partner attitudes.

**Societal level factors**: influence the acceptability and expectations about breastfeeding and provide the background or the context in which mothers’ feeding practices occur. These include cultural norms regarding breastfeeding, child feeding, and parenting; the role of women in society, including how working outside the home is valued; the extent to which men’s social role includes support for breastfeeding mothers; the extent to which exposing breasts for feeding is complicated by cultural norms regarding sexuality; and the economic importance of products such as breast milk substitutes and complementary foods in the food system.

**Dependent Variables**: practice of exclusive breast feeding.

**Independent variables**: such as socio-demographic, awareness of exclusive breastfeeding and health service related characteristics.

### Statistical analysis

Data was entered through Epi Info 7 for Dos version 3.5.1 then exported to Statistical Package for the Social Sciences (SPSS) software windows version 20 for analysis. Descriptive statistics was to determine the prevalence of exclusive breast feeding. Proportion was compared by exclusive breast feeding using correlation of independent variables. To identify associated factors, first a bivariate logistic regression was performed for each independent variable with the outcome of exclusive breast feeding. Finally, multivariable logistic regression was implemented to determine independent predictors of exclusive breast feeding. All tests were *P* < 0.05 considered as statistically significant.

## Results

A total of 420 women participated in the study. The age range of mothers considered in the study was 15–49 years, which is a childbearing age range. The mean (±Standard deviation) age of the mothers was nearly 27 years (0.9).The age range of children considered in this study was 0–6 months, which is an optimal age range for exclusive breast feeding. The majority of children 256(61 %) were between 4 and 5 months of age. The maximum and minimum numbers of children were found in the two extreme months of age. The mean (± standard deviation) age of the children was 2.5 months (0.72). The majority of the mothers 417(99.3 %) were house wives/unemployed and the majority were Muslim religion (Table 2).

**Table 1:** Summary of sample size calculation based on different predictors

Sr. No	Predictor	Odds of EBF	Frequency of unexposed group	% of CI	Power	SS cases	SS control	Sample size	10 % non-respondent	Total sample size
1	Pre-lacteal feeding	2	41.9 %	95	80	138	151	289	28.9	318
2	Place of delivery	4.5	32.1 %	95	80	33	37	70	7	77
3	Type of delivery	2.25	40 %	95	80	102	113	215	21.5	237

**Table 2:** Frequency distribution of selected socio demographic characteristics among mother-child paired subject groups of the study; Halaba special woreda, SNNPR Ethiopia, 2014

Socio demographic characteristics of mother-child paired study subjects (*n* = 420)		
Variables	Numbers	Percent (%)
Age of mother in years		
15–19	7	1.7
20–24	73	17.4
25–29	164	39.0
30–34	143	34.0
≥35	33	7.9
Age of child in months		
0–1	56	13.3
2–3	108	25.7
4–5	256	61.0
Sex of child		
Male	205	48.8
Female	215	51.2
Relation of mother to head of household		
Wife	417	99.3
Other than wife	3	0.7
Religion		
Muslim	413	98.3
Christian	7	1.7
Ethnicity		
Halaba	393	93.6
Kambata	17	4.0
Hadiya	1	0.2
Oromo	4	1.0
Silte	5	1.2
Attended formal school		
Yes	106	25.2
No	314	74.8
Marital status		
Single	5	1.2
Married (active)	414	98.6
Divorced	1	0.2
Occupation		
Student	3	0.7
Worker	9	2.2
House wife/unemployed	408	97.1
Average monthly income		
0–100 Birr	44	10.5
101–500 Birr	225	53.6
500 ≥ Birr	75	17.9
Don’t know	74	17.6

About 82 and 29 % of the mothers had followed antenatal and postnatal care while they were pregnant and after birth of the index child respectively. The mean (±standard deviation) number of times antenatal follow-up was 3.13(1.23) throughout the gestational age.

About 204(48.6 %) of the mothers initiated breast feeding for the children with in one hours after birth, the rest 216(51.4 %) of mothers started breast feeding after one hours and 24 h. Regarding the frequency of breast feeding, mothers had asked whether their last child was breast fed on demand, schedule or on both.

A total of 296(70.5 %) of mothers and 388(92.4 %) of mothers have practicing exclusive breast feeding and have awareness on exclusive breast feeding respectively. Regarding still breast feeding has 419(99.8 %) and also not practicing pre-lacteal feeding has 350(83.5 %) (Table 3).

**Table 3:** Frequency distribution of exclusive breast-feeding and health service related variables among mother-child paired subject groups of the study; Halaba sp.woreda, SNNPR Ethiopia, 2014

Distribution of exclusive breast feeding and health Service related variables (*n* = 420)		
Variables	Number	Percent (%)
ANC follow-up		
Yes	345	82.1
No	74	17.6
Don’t Know	1	0.2
PNC Follow-up		
Yes	124	29.5
No	292	69.5
Don’t Know	4	1.0
Advised about EBF		
Yes	184	43.8
No	229	54.5
Don’t Know	7	1.7
Place of birth		
Health facilities	88	21.0
Home	332	79.0
Initiation of breast-feeding		
Immediately/within 1 h	204	48.6
After 1 h	216	51.4
Frequency of breast-feeding (*n* = 418)		
≥8 times per day	309	73.6
<8 times per day	109	26.0
Exclusive breast-feeding		
Yes	296	70.5
No	124	29.5
Awareness about exclusive		
Yes	388	92.4
No	32	7.6
Still breastfeeding		
Yes	419	99.8
No	1	0.2
Pre-lacteal feeding		
Nothing	350	83.3
Other than breast milk	70	16.7

A total of 106(72 %) age of mothers between 24 and 28 years old have practicing exclusive breast feeding as compared to age of mothers between 29 and 33 years of age. Among age of children between 0 and 6 months, mothers who have child age of 0–1 months 43(78.2 %), 2–3 months 78(71.6 %) and 4–5months old practicing 175(68.4 %). This show that the mothers who have child age of 0–1 months practices exclusive breast feeding higher than the other age group 2–3 months and 4–5 months, which is as the age increase the practice of exclusive breast feeding practice decrease (Fig. 4). As compared to mothers who have female child to male, mothers who have male child practicing exclusive breast feeding almost the same, which is 145(70.7 %) and 151(70.2 %) (Table 4).

**Fig. 1:**
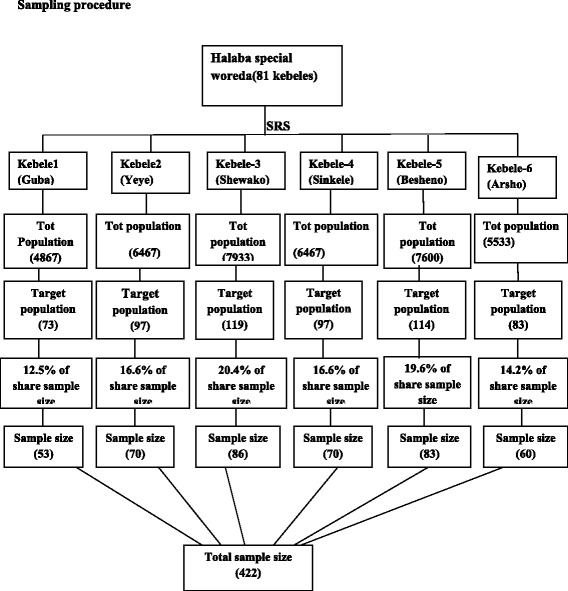
Schematic presentation of sampling technique

**Fig. 2:**
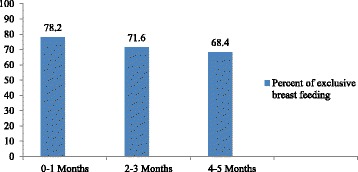
Month specific life-time exclusive breast feeding practice (*n* = 420)

**Fig. 3:**
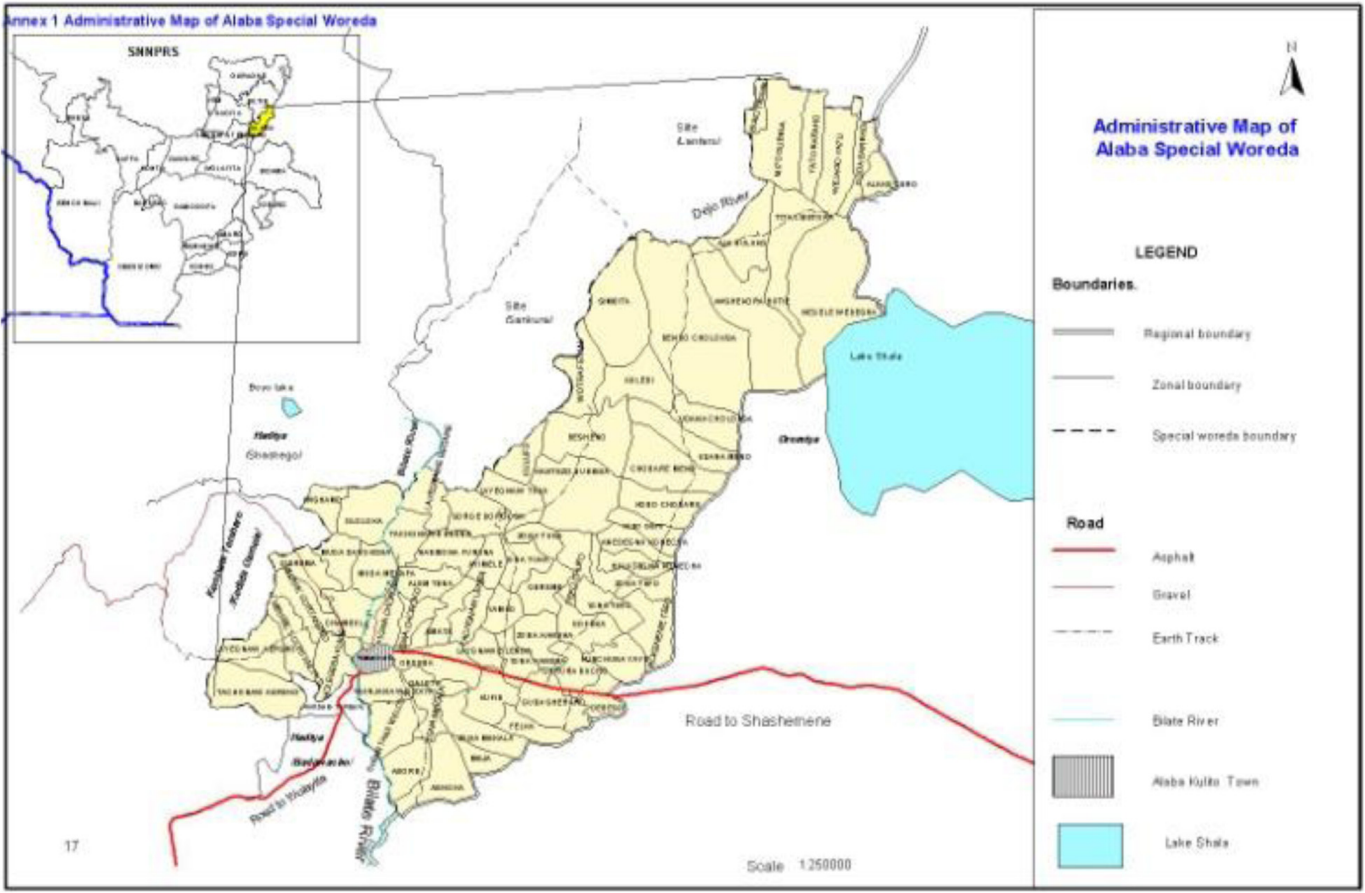
Area map of Halaba special woreda. Source: Halaba specil aworeda annual health report for the year 2005 E.C [9]

**Fig. 4:**
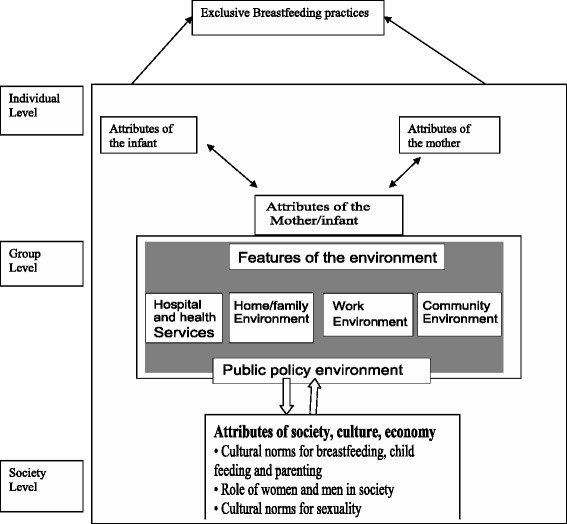
A conceptual framework of predictors that influence exclusive breastfeeding practice [28]

**Table 4:** Exclusive breast-feeding practices among mothers of children aged less than six months of age, by selected socio demographic and health service related variables; Halaba Special woreda, SNNPR Ethiopia, 2014

Exclusive breastfeeding practices (*n* = 420)				
Variables	Yes	No	Crude OR (CI)	Adjusted OR (CI)
	Number (%)	Number (%)		
Age of mother in years				
15–19	5(71.4)	2(28.6)	1.0(0.17–6.5)	
20–24	51(69.9)	22(30.1)	1.2(0.46–2.8)	
25–29	121(73.8)	43(26.2)	0.95(0.4–2.2)	
30–34	95(66.4)	48(33.6)	0.74(0.29–1.84)	
≥35	24(72.7)	9(27.3)	1.00	
Age of child in months				
0–1 Months	43(78.2)	12(21.8)	0.65(0.33–1.28)	
2–3 Months	78(71.6)	31(28.4)	1.66(0.79–3.52)	
4–5 Months	175(68.4)	81(31.6)	1.00	
Sex of child				
Male	145(70.7)	60(29.3)	0.98(0.64–1.49)	
Female	151(70.2)	64(29.8)	1.00	
Attended formal school				
Yes	87(82.1)	19(17.9)	2.3(1.29.4.14)*	6.3(1.3–30.9)*
No	209(66.6)	105(33.4)	1.00	1.00
Marital status				
Married	294(71)	120(29)	4.9(0.88–27.1)	
Other than Married	2(33.3)	4(66.7)	1.00	
Occupation				
House wife	287(70.9)	118(29.1)	1.62(0.56–4.65)	9.9(1.01–98.6)*
Worker, other	9(60)	6(40)	1.00	1.00
Average monthly income				
0–100 Birr	28(63.6)	16(36.4)	0.37(0.2–0.8)*	
101–500 Birr	176(78.2)	49(21.8)	0.18(0.1–0.3)*	3.6(1.9–6.8)*
500 ≥ Birr	62(82.7)	13(17.3)	0.14(0.06–0.3)*	4.8(2.0–11.5)*
Don’t know	29(39.2)	45(60.8)	1.00	1.00
ANC follow-up				
Yes	258(74.6)	88(25.4)	2.7(1.6–4.5)*	1.9(1.05–3.6)*
No, Don’t know	38(51.4)	36(48.6)	1.00	1.00
PNC Follow-up				
Yes	98(79.8)	25(20.2)	1.97(1.19–3.25)	2.2(1.25–3.87)*
No, Don’t know	197(66.5)	99(33.5)	1.00	1.00
Advised about EBF during ANC, Delivery &PNC				
Yes	166(90.2)	18(9.8)	6.9(1.12–40.99)*	1.9(1.1–3.4)*
No	126(55)	103(45)	1.09(0.24–4.98)	
Don’t Know	4(57)	3(43)	1.00	1.00
Place of birth				
Health facilities	66(75)	22(25)	1.33(0.78–2.27)	
Home	230(69.3)	102(30.7)	1.00	
Initiation of breast-feeding				
Immediately/within 1 h	149(72.3)	57(27.7)	1.19(0.77–1.85)	3.8(2.4–6.1)*
After 1 h	147(68.7)	67(31.3)	1.00	1.00
Frequency of breast-feeding (*n* = 418)				
≥8 times per day	69(63.3)	40(36.7)	0.64(0.4–1.05)	
<8 times per day	225(72.8)	84(27.2)	1.00	
Awareness about EBF				
Yes	287(74)	101(26)	7.3(3.08–17.6))*	8.2(3.34–20)*
No	9(28.1)	23(71.9)	1.00	1.00
Pre-lacteal feeding				
Nothing	251(71.7)	99(28.3)	1.4(0.82–2.42)	
Other than breast milk	45(64.3)	25(35.7)	1.00	
Total	296(70.5)	124(29.5)		

*It describe the significant association in the crud and adjusted OR

After an adjustment, using logistic regression model, mothers who have house wife/unemployed, attended formal school and average monthly incomes 101Birr up to 500 Birr and above and equal 500 Birr showed significant association with the practice of exclusive breast feeding (Table 4).

After an adjustment was made,using logistic regression model, antenatal follow up(AOR = 1.9;95 % CI,1.05–3.6), postnatal follow up (AOR = 2.2;95 % CI,1.25–3.8), initiation of breast feeding immediately within one hour (AOR = 3.8;95 % CI,2.4–6.1), advice about EBF during ANC,PNC and delivery (AOR = 1.9;95 % CI,1.1–3.4) and awareness about exclusive breastfeeding (AOR = 8.2;95 % CI,3.34–20) established significant association with in the outcome exclusive breast feeding practice.

## Discussion

The overall prevalence of exclusive breastfeeding in the study subject was 70.5 %, with 95 % CI (66.2–74.8) which is comparable with the result from previous study done in Oromia region Goba district (71.3 %), Madagascar (70 %), and Zambia (74 %), But this finding is higher than the findings in in Harare (51.9 %), the national exclusive breastfeeding prevalence in Ethiopia (52 %), Tanzania (58 %), and Bangladesh (36 %) [7, 8]. These results showed wide variation of exclusive breastfeeding prevalence between and within countries and over time. This is due to different non- governmental organization in collaboration with woreda health office implementing Infant and young child feeding (IYCF) and community based nutrition (CBN) intervention in study areas. The other point is different methodologies for estimating the rate of exclusive breastfeeding may also influence the results [9].

About 68 % of infants between 4 and 5 months were breast fed exclusively. This finding is higher than the study was done in Oromia Goba district which is (17 %),the Ethiopian national months –specific exclusive breast feeding aged 4–6 months (32 %), a survey conducted on the practice of infant feeding and the influencing factors in United Arab Emirates, showed that (46 %) and [6, 8, 19]. As the age of children approached 6 months, the percentage of exclusive breastfeeding decreased. This might be due to the fact that postpartum care is traditionally given in the first few months after birth where mothers remain at home; creating a chance to exclusively breast feed their infant. The other possible reason might be that mothers introduce pre-lacteal feeding for their infant due to the assumption that breast milk alone would not satisfy their needs as the infants gets older [8, 22].

This study has revealed that housewife/unemployed mothers are predictors of exclusive breastfeeding, which is consistent with the findings of several studies. This might be explained by the fact of less maternity leave (two months after delivery in our context), which makes workers mothers have less opportunity to stay at home. Mothers also may have to leave their babies to search for a job. These findings call for policy arguments, as well as the extension of maternity leave to encourage mothers to exclusively breast their babies to improve child health outcome [6, 8, 9, 18]. The majority of the mothers 417(99.3 %) were house wives/unemployed and the majority were Muslim religion (Table 2). This might have positive impact on exclusive breast feeding by providing sufficient time for infants and mother.

Children from mothers who were average monthly incomes 101Birr up to 500 Birr and above showed more likely to practice exclusive breastfeeding to those mothers who were average monthly incomes less than 101 Birr. This is similar to the study conducted in infant and young child feeding practice among mothers living in Harar, Ethiopia and in USA [15, 23, 25]. This might be due to the average monthly incomes of the mother’s mater the health care seeking behavior of the mother to visit the health facility for different health related services. Accordingly, during the mother visited to health facilities the health provider at health facilities have the opportunities to provide the information related to exclusive breast-feeding.

Children from mothers who attended formal school are more likely to practice breast feeding to those mothers who were not attended. This can be explained by the mother who attended formal school has the opportunities to exposed herself for information related to exclusive breast feeding through different kinds of media channel likes; posters, family health cards and other electronic information and education martials that might be influence the exclusive breast feeding practice [4, 24, 30].

Children from mothers who attended ANC are more likely to practice exclusive breastfeeding to those mothers who were not attended ANC. This might be due to during the visit the health workers advised the mothers about exclusive breast feeding and others health related services [9].

Children from mothers who were attended PNC are also more likely to practice exclusive breastfeeding to those mothers who were not attended PNC. This also due to the mothers advised about exclusive breast feeding practice by health providers, while they were visited health facilities [4, 5].

Advice about exclusive breastfeeding during ANC, PNC, and delivery are more likely to practice exclusive breasting to those mothers who were not. This is might be due to availability of procedure manual and guideline and training for most staff on maternal infant and young child feeding might have contributed to higher EBF knowledge and counseling skills among health workers in study area [1, 2].

Children from mothers who were exposed for early initiation of breastfeeding immediately within one hour are more likely to practice exclusive breast feeding as compared with those mothers above one hour and a day. This might be due to the health providers helped mothers to initiate breastfeeding within one hour of birth while the mother attended delivery at health facilities [3, 9-20].

Children from mothers who had awareness about exclusive breastfeeding practice were more likely to practice exclusive breastfeeding to those who were not awareness about exclusive breastfeeding. This is due to the mother acquired awareness about the benefit of exclusive breastfeeding during when she visited the health facility, visited at home by health extension workers and strong community based nutrition program by different partners that might be influenced the prevalence of exclusive breastfeeding, which is similar with the study conducted in Western Tanzania and Nigeria [3, 9-20]. So that intensified efforts are needed to make sure that women have universal access to current information regarding exclusive breastfeeding and its advantage during the mothers visited health facility in major action points.

### Limitation of the study

It has a limitation to formulate a causal association, as to how and when the associations are established, since the study design was a cross sectional which is studies are carried out at one time point or a short period. The fact that this study did not assess individual factors, including knowledge rather than awareness and attitude of mother, as well as variables related to family and peers deeply

## Conclusion

In this study, the prevalence of exclusive breastfeeding for the age group 0–6 months (70.5 %) were below the World Health Organization infant and young child feeding recommendation (90 %). The practice of exclusive breastfeeding mothers who participate in the study was influenced by the awareness of mothers about the benefit of exclusive breastfeeding, antenatal follow up, postnatal follow up, early initiation of breast milk within one hour and advice about EBF during ANC, PNC and delivery. Furthermore, average monthly income of the family, education and unemployment revealed that the predictors of EBF. Strategies that target improving awareness among women at health facility and community level help to improve exclusive breastfeeding. Working mothers were more likely not to exclusively breastfeeding their babies. So that promotion of EBF for working mothers through working environment recommended.

## Abbreviations

AIDS: Acquired Immune Deficiency Syndrome
ANC: Antenatal Care
AOR: Adjusted odds ratio
CBN: Community Based Nutrition
CI: Confidence Interval
E.C: Ethiopian Calendar
EBF: Exclusive breast feeding
EDHS: Ethiopia Demographic Health Survey
ETB: Ethiopia Birr
HIV: Human Immune Virus
IYCF: Infant and Young Child Feeding
MPH: Master of Public Health
MTCT: Mather to Child Transmission
OR: Odds Ratio
PNC: Postnatal Care
SNNPR: Southern Nation Nationality People Region
SPSS: Statistical Package for the Social Sciences
SRS: Simple Random Sampling
UNICEF: United Nation Children’s Fund
WHO: World Health Organization
